# Reducing the cesarean delivery rates for breech presentations: administration of spinal anesthesia facilitates manipulation to cephalic presentation, but is it cost saving?

**DOI:** 10.1186/2045-4015-3-5

**Published:** 2014-02-24

**Authors:** Carolyn F Weiniger, Paul S Spencer, Yuval Weiss, Gary Ginsberg, Yossef Ezra

**Affiliations:** 1 Department of Anesthesiology and Critical Care Medicine Hadassah-Hebrew, University Medical Center, Ein Kerem, Jerusalem 91120, Israel; 2 Department of Anesthesia, Stanford School of Medicine, Stanford, CA, USA; 3 Department of Electrical Engineering & Computer Sciences, University of California at Berkley, California, USA; 4 Hospital administration, Hadassah-Hebrew University Medical Center, Ein Kerem, Israel; 5 Department of Medical Technology Assessment, Ministry of Health, Jerusalem, Israel; 6 Department of Obstetrics & Gynecology, Hadassah-Hebrew University Medical Center, Ein Kerem, Jerusalem, Israel

**Keywords:** Breech, Cesarean delivery, Costs, Neuraxial blockade, Spinal

## Abstract

**Background:**

External cephalic version (ECV) is infrequently performed and 98% of breech presenting fetuses are delivered surgically. Neuraxial analgesia can increase the success rate of ECV significantly, potentially reducing cesarean delivery rates for breech presentation. The current study aims to determine whether the additional cost to the hospital of spinal anesthesia for ECV is offset by cost savings generated by reduced cesarean delivery.

**Methods:**

In our tertiary hospital, three variables manpower, disposables, and fixed costs were calculated for ECV, ECV plus anesthetic doses of spinal block, vaginal delivery and cesarean delivery. Total procedure costs were compared for possible delivery pathways. Manpower data were obtained from management payroll, fixed costs by calculating cost/lifetime usage rate and disposables were micro-costed in 2008, expressed in 2013 NIS.

**Results:**

Cesarean delivery is the most expensive option, 11670.54 NIS and vaginal delivery following successful ECV under spinal block costs 5497.2 NIS. ECV alone costs 960.21 NIS, ECV plus spinal anesthesia costs 1386.97 NIS. The highest individual cost items for vaginal, cesarean delivery and ECV were for manpower. Expensive fixed costs for cesarean delivery included operating room trays and postnatal hospitalization (minimum 3 days). ECV with spinal block is cheaper due to lower expected cesarean delivery rate and its lower associated costs.

**Conclusions:**

The additional cost of the spinal anesthesia is offset by increased success rates for the ECV procedure resulting in reduction in the cesarean delivery rate.

## Background

Cesarean delivery is recommended by national guidelines for the breech presenting fetus (3-4% of pregnancies) [1,2]. These same national guidelines recommend the availability of an external cephalic version (ECV) service; potentially enabling attempted vaginal delivery. Unfortunately, ECV is infrequently performed, hence 98% of breech presentations are delivered surgically [3,4]. Potential reasons for poor uptake of ECV include low ECV success rates, lack of physician referral and unwillingness of patients to undergo the procedure [3,5,6].

Immediate hospital and health fund costs of cesarean delivery are higher than vaginal delivery [7,8]. A computer-based model of ECV trial versus scheduled cesarean delivery found that ECV is cost-effective above a success threshold of 32% [7]. Addition of neuraxial blockade for ECV generates a hospital cost benefit if the ECV success rate is increased 11% above a baseline of 38% without neuraxial blockade [9]. However the study showing these data combined anesthetic (increased ECV success rate) and analgesic (little effect on ECV success) doses in their analysis; potentially muting the findings.

In ideal circumstances, vaginal delivery of a cephalic presentation is the safest route for both mother and neonate [10,11]. Uptake of ECV can be encouraged if success rates of ECV are maximally high; for example through use of neuraxial blockade [12-14].

The current study aims to determine whether the additional cost to the hospital of spinal anesthesia is offset by increased success rates for the ECV procedure; through decreased cesarean delivery rates.

## Methods

The study was carried out in a tertiary hospital in the Labor and Delivery suite using financial data obtained from 2008 in New Israeli Shekels (NIS) and costs were adjusted to 2013 NIS using the consumer price index. The conversion factor used is 1.3336229. We present the costs to the hospital. Fixed equipment costs were calculated as a unit cost for the expected lifetime of the item. The fixed costs, for expenses such as hospital bed and multi-use equipment (blood pressure monitor, fetal heart rate monitor, and ultrasound) were calculated as a 20% overhead according to the accepted Israeli Ministry of Health method. Disposables include gloves, spinal needles, drugs, and sterile pack for performing anesthesia of surgery. Personnel include costs of all the staff (obstetricians, midwives and anesthesiologists) involved in each procedure: vaginal delivery, cesarean delivery and ECV with and without spinal anesthesia. Ethical approval was not required for this cost analysis study.

Current practice for management of breech presentation in our institution comprises performing ECV, or elective cesarean delivery. In the case of successful ECV - vaginal delivery may be attempted.

We systematically searched Cochrane, Medline and Web of Science databases from 1990 to January 2013 using key words; “breech; external cephalic version; ECV; anesthesia; analgesia; spinal; epidural; neuraxial; version; fetal”. We excluded manuscripts based upon title and abstract content. We manually searched the bibliography of relevant manuscripts. Using this search strategy we identified 6 randomized controlled trial publications using neuraxial blockade (comprising analgesia or anesthesia doses) for ECV and 3 cost analyses, and used these data to calculate our probability of ECV success with spinal anesthesia and delivery outcomes [7-9,15-20].

### Data analysis

For each delivery management pathway the individual itemized costs for each major variable (fixed costs, disposables, and manpower) were entered as a separate item into an Excel worksheet (Microsoft Office 2000). Thus each delivery management pathway (vaginal delivery, cesarean delivery, spinal anesthesia) is presented as a unit total cost.

We pooled data from our two randomized controlled trials with an overall success rate of 76.1% for ECV using spinal anesthesia versus 44.8% ECV success rate without anesthesia – obtained from 134 enrolled patients (64 multiparas and 70 nulliparas) [19,20]. For our model, we used a vaginal delivery rate derived from the total rate of vaginal cephalic delivery following successful ECV in our pooled study population: 68 successful cephalic vaginal deliveries from 81 successful ECVs (84%). Thus 16% of successful ECV underwent cesarean delivery. This cesarean delivery rate was within the range reported among 206,909 deliveries between 2002–2008; the cesarean delivery rate for cephalic presentation intrapartum was 12.8%, and for attempted vaginal delivery after labor induction was 21.1%. Other studies reported a cesarean delivery rate after successful ECV of 16-26% [21,22].

Confidence intervals were calculated for the management pathways rates for cesarean and vaginal delivery using WinPepi version 11.15; using sample sizes from the derivation data [19,20].

## Results

Among 150,000 deliveries per year in Israel, up to 4% are breech presentation – 6000 breech deliveries/year. In the case that 76% of ECV performed are successful using spinal anesthesia this would result in 4,560 potential vaginal deliveries. Of these, 16% may undergo cesarean, but 3830 (84%) additional vaginal deliveries may occur.

The sum of the number of items and the costs per possible procedure performed are reported in Table 1. Cesarean delivery is the most expensive option, 11670.54 NIS, and vaginal delivery costs 4110.23 NIS. Successful ECV under spinal anesthesia with subsequent vaginal delivery costs 5497.2 NIS. Addition of spinal anesthesia for the ECV procedure costs 426.76 NIS. Using the costs presented in Table 1, maximum savings can be calculated: 8,321,820 NIS for spinal anesthesia for ECV of 6000 breech presentation cases, in addition to the delivery costs (3830 vaginal deliveries = 15,742, 181 NIS and 2170 cesarean deliveries = 25, 325, 072 NIS) generating a total costs of 49, 389, 07 NIS. If all 6000 breech presentations were delivered by cesarean delivery without spinal anesthesia, the cost is 70, 023 240 NIS, generating a maximum possible saving of 20, 634, 167 NIS nationally in Israel per year.

**Table 1:** Major variables reported per individual management option

**Total cost (NIS)**	**Personnel cost (NIS)**	**Disposable cost (NIS)**	**Fixed cost (NIS)**	**No. items total per procedure**	**Procedure**
11670.54	4076.89	1298.95	6294.70	77	Cesarean delivery
4110.23	1824.40	250.72	2035.11	39	Vaginal delivery
960.21	533.45	17.34	409.42	10	ECV
1386.97	808.18	169.37	409.42	18	ECV plus Spinal block

Key: Costs sourced in 2008 were adjusted to 2013 NIS price levels using the consumer price index. The conversion factor used is 1.3336229. (Central Bureau of Statistics, Price Statistics Monthly - September 2013. Jerusalem). Costs were sourced from hospital budget itemizations.

ECV = external cephalic version, NIS = New Israeli Shekel.

The highest individual costs were for manpower for vaginal delivery, cesarean delivery and ECV. It was calculated that nursing care hours required were 14 per vaginal delivery, 4 per ECV procedure, 5.5 for cesarean delivery and physician hours would be 1.5, 2, and 3.5 for vaginal delivery, ECV and cesarean delivery, respectively. Expensive fixed costs for cesarean delivery include operating room trays and postnatal hospitalization (minimum 3 days).

**Figure 1:**
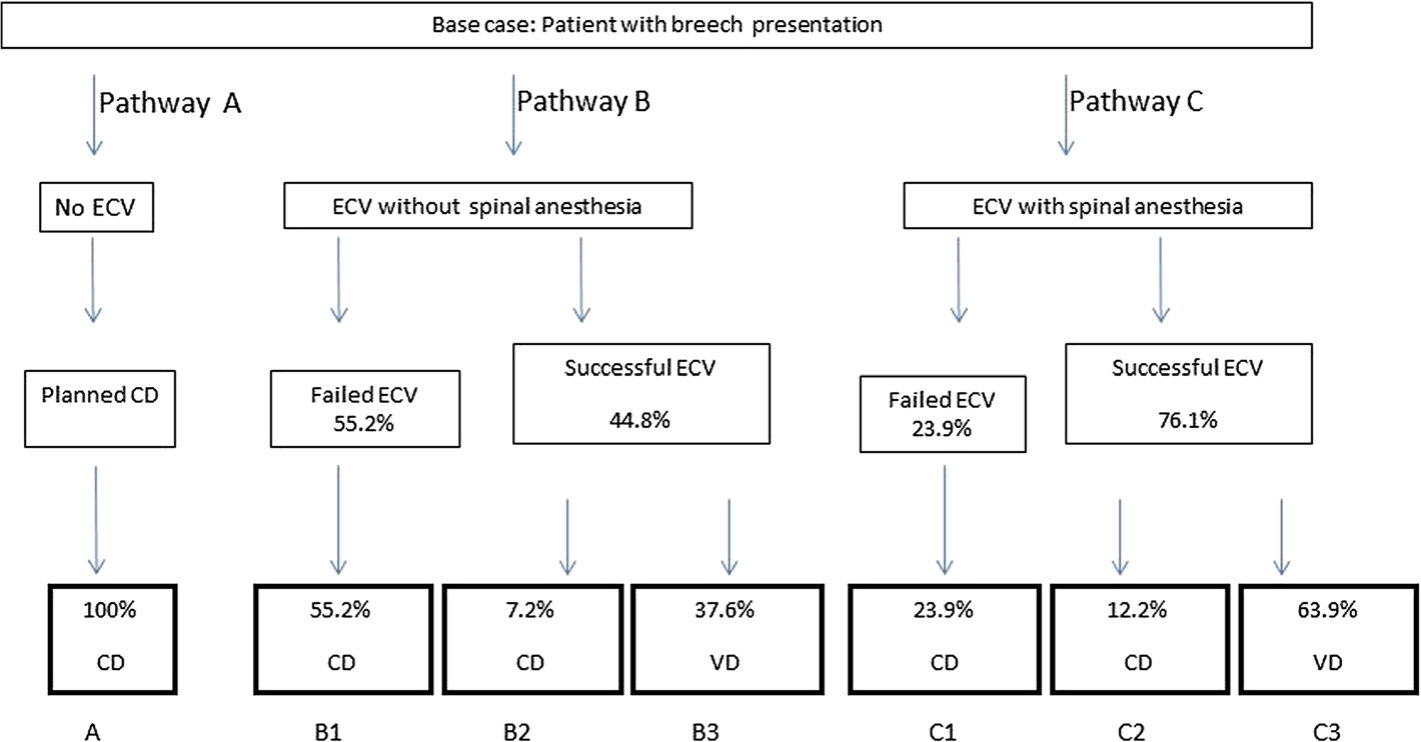
**Potential delivery pathways for the base case patient with breech presenting fetus.** The patient with breech presenting fetus can go directly to cesarean delivery, or undergo ECV, either with (Pathway **B**) or without (Pathway **C**) spinal anesthesia. ECV success rates with and without spinal anesthesia were taken from our previous publications [19,20]. Pathway **A** (no ECV) shows 100% CD rate, despite the possibility of vaginal breech delivery or spontaneous conversion to cephalic presentation. For pathways B2/B3 and C2/C3, following successful ECV, a cesarean delivery rate of 16% is used, based upon calculated data from Table 3. Costs associated with each pathway are presented in Table 3. VD = vaginal delivery, CD = cesarean delivery.

**Figure 2:**
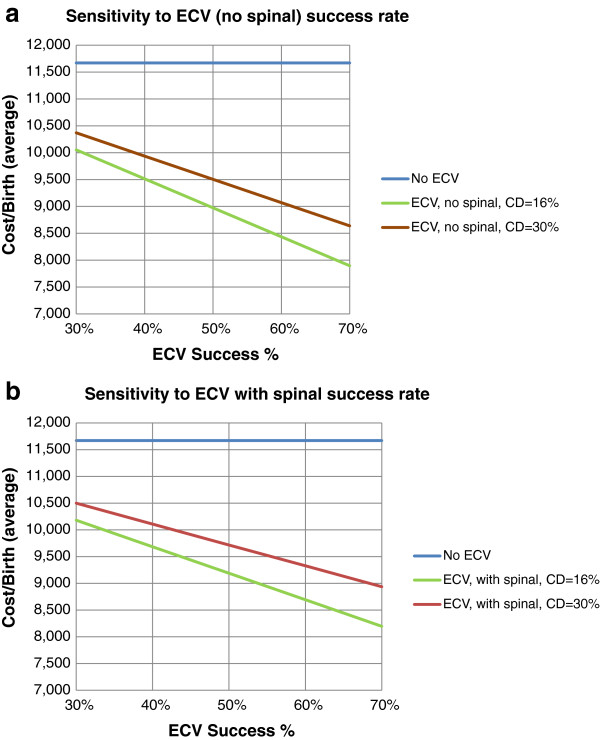
**Reported ECV success rates range from 30-70%; higher ECV success generate higher vaginal delivery rates - decreasing costs.** Figure 2**a** shows ECV success rates ranging from 30-70% without spinal anesthesia and Figure 2**b** shows ECV success rates ranging from 30-70% with spinal anesthesia. Both show costs for a 16% and 30% cesarean delivery rate. If the prevailing cesarean delivery rate increases from 16% to 30%, the cost savings from ECV success rates falls.

Six randomized controlled trials (RCTs) report success rates for ECV, using neuraxial blockade compared to control, Table 2[15-20]. The delivery rates in Figure 1 were retrieved from our previous studies for vaginal and cesarean delivery (combined multiparas and nulliparas) [19,20]. Previous RCTs using anesthesia for successful ECV reported a combined cesarean delivery rate of 16.3% [15,16,19,20]. When analgesia, rather than anesthesia is considered for ECV, successful cases have a reported cesarean delivery rate of 14% [17,18]. Table 3 is based upon the pathway rates presented in Figure 1. The costs associated with breech delivery are reduced as ECV success increases across a range of rates, and costs are additionally dependent on the cesarean delivery rate following successful ECV, Figure 2a and b.

**Table 2:** Vaginal delivery rates following ECV with neuraxial block for breech presentation (analgesia or anesthesia dose) in previously published randomized trials

**Authors**	**Analgesia/Anesthesia**	**Effect size in treatment group**	**Effect size in control group**	**Vaginal cephalic delivery in treatment group**	**Vaginal cephalic delivery in control group**
Dugoff [22]	Analgesia	22/50 (44%)	22/52 (42%)	16/50^‡^ (32.0%)	25/52^‡^ (48.1%)
Mancuso [20]	Anesthesia	32/54 (59%)	18/54 (33.3%)	28/54^a^ (51.9%)	13/54^a^ (24.1%)
Schorr [19]	Anesthesia	24/35 (69%)	11/34 (32.4%)	23/35 (65.7%)	7/34 (20.6%)
Sullivan [21]	Analgesia	22/47 (47%)	15/48 (31.3%)	17/47 (36.2%)	12/48 (25%)
Weiniger [16]	Anesthesia	24/36 (67%)	11/34 (32.4%)	16/36 (44.4%)	8/34 (23.5%)
Weiniger [15]	Anesthesia	27/31 (87%)	19/33 (57.6%)	25/31^b^ (80.%)	19/33^b^ (57.6%)

adata presented are vaginal cephalic delivery only.

Effect size = ECV success rate.

^b^excludes vaginal breech delivery and repeat ECV.

^‡^Following unsuccessful ECV five patients overall had spontaneous version to cephalic presentation with vaginal delivery.

**Table 3:** **Costs for management pathways, derived from decision tree, Figure**1

**Pathways for breech management**	**Management procedures (ECV with/without spinal, subsequent VD or CD)**	**Unit cost NIS**	**CD/VD rates after ECV **[19]**,**[20]**Relative probability (95% CI)**	**Relative cost NIS (CD rate 16% after successful ECV)**
*Pathway A*				
No ECV	CD	11670.54	100%	
			*Total pathway A*	*11670.54*
*Pathway B*				
B1	ECV (failure), CD	12630.75	55.2% (0.40-0.64)	6972.17
B2	ECV (success), CD	12630.75	7.2% (0.03-0.16)	909.41
B3	ECV (success), VD	5070.44	37.6% (0.26-0.49)	1906.49
			*Total pathway B*	*9988.07*
*Pathway*				
C1	ECV + spinal, CD	13057.51	23.9% (0.15-0.35)	3120.74
C2	ECV + spinal, CD	13057.51	12.2% (0.06-0.21)	1593.02
C3	ECV + spinal, VD	5497.2	63.9% (0.52-0.75)	3512.71
			*Total pathway C*	*8226.47*

Key: Costs are derived from Table 1 unit costs and% of each pathway occurring.

CD = cesarean delivery, VD = vaginal delivery, ECV = external cephalic version, CI = confidence interval.

Pathway A = no ECV, Pathway B = ECV without spinal anesthesia, Pathway C = ECV with spinal anesthesia.

For CI calculation, the denominator is 67 (Table 3) [19,20].

## Discussion

In the current study we found that complications and quality of life considerations aside, adoption of policy of ECV with spinal anesthesia engenders hospital cost saving benefit through increased ECV success rates and subsequent decreased cesarean deliveries. The hospital cost of 426.76 NIS per anesthetic for ECV generates a cost saving of 1761.27 through decreased cesarean delivery - if the vaginal delivery rate following successful ECV is 84%. For the 6,000 annual breech presenting births in Israel, this could save the hospital providers over 20 million NIS per year if ECV were performed under anesthesia for all breech presentation, and if ECV is maximally successful and cesarean delivery rates are minimal.

Several studies report that adopting a policy of ECV for the breech presenting fetus could result in a significant cost saving, in particular when neuraxial blockade is used [7-9]. Previously reported success rates of ECV, range from 30-70% [23,24]. The current study considers ECV performed using spinal anesthesia, which significantly increases the ECV success rate to over 67%. The success rates from ECV with neuraxial blockade vary considerably depending on whether an anesthetic or analgesic dose was used. Carvalho et al., demonstrated cost benefit for ECV using a combination of data from anesthesia and analgesia studies despite the latter being less effective at influencing ECV success rates [9]. Anesthetic rather than analgesia doses are more effective [25].

The ultimate aim of ECV is to reduce the cesarean delivery rates; breech cesarean delivery accounts for 25% of elective cases [26]. Following one cesarean delivery for breech presentation, subsequent cesarean delivery is expected in 90% of cases [27]. We calculated data based upon a 16% cesarean delivery rate following ECV success. Other studies report a wide range of cesarean delivery rates following successful ECV, from 16.4-50% [21,28-31]. A lower overall cesarean delivery rate in a specific population undergoing vaginal delivery generates greater potential cost benefit using neuraxial block for ECV. Spinal anesthesia has a greater effect on the base case cost over the range of possible ECV success rates (30-70%), if the prevailing cesarean delivery rate is 16%. When the cesarean delivery rate is 30%, the cost differential between the ECV success ranges is less pronounced.

The current study micro-costed the hospital purchasing price of every item involved in the costs of performing each procedure (fixed, equipment, and personnel). Hence the actual costs of ECV, spinal block, normal delivery, and cesarean delivery, were calculated using the known itemized costs as has been done previously [8,9]. Other previous studies have used national department of health figures for calculating costs which are based on a national tariff or reimbursement fees, rather than actual itemized costs [23,24]. Costs differ greatly among countries. The cost of ECV in the USA is significantly higher than in the UK or in our current Israeli study [7,8,15,32]. The key factor in this cost variance is salary differences between the countries [33]. Recent data from the USA reported that ECV with neuraxial block costs 1221$ and without neuraxial block 1087$ in 2010 [9]. This is almost four times the Israeli 2008 costs, however the same authors reported their ECV cost without neuraxial block in 2007 was only slightly lower, 1024$. James et al. in a study from the UK, micro-costed each procedure involved in the ECV pathway, similar to the current study, and calculated ECV to cost approximately 284$ (1136 NIS), only slightly higher than our local estimate [8]. Their study reported ECV but without neuraxial block, which may have significantly increased ECV success rates, thus providing a more remarkable cost saving. A cesarean delivery in the UK was also more expensive at 2930$ compared with 2500$ in Israel. The UK study did not factor in the cost of neuraxial block, and the average ECV success rate was only 50%, less than that expected with use of spinal anesthesia in the current study. Since Ministry of Health imposes hospital costs in Israel, ours may reflect those of other Israeli institutions. Although ECV is recommended by the American College of Obstetrics and Gynecologists (guidelines adopted in Israel), national ramifications of our study findings should consider that ECV practice may differ among institutions, as do cesarean rates.

A further cost consideration in the US is actual re-imbursement to the hospital. The low USA reimbursement costs calculated on Relative Value Units (RVUs) for ECV (1.71) compared with cesarean delivery (16.33) may not encourage practice [34]. Personnel costs are considerably higher in the US compared with UK and Israel, albeit data for the UK are from 1997 [8,9]. Attempts to favor vaginal delivery through reimbursement have not curtailed the increase in cesarean delivery [4]. Hospital costs may be higher or lower than actual reimbursement costs, depending on how the hospital calculates their actual ECV costs.

Another important factor to consider is quality adjusted life years (QALYs) resulting from a particular delivery management plan. Factors affecting QALYs for cesarean delivery include maternal wound infection and duration of disability from surgical scar. Following normal vaginal delivery, relevant QALYS include immobility following perineal tears. The current study did not consider sufficient patients to calculate QALYs. However previous data suggest that normal vaginal delivery is less costly in terms of QALYs than cesarean delivery [7].

The data we present focus on hospital savings and ignore other potential costs benefits to the patient, family or health fund. The success rates of ECV under neuraxial block may differ between institutions, which would impact the savings found [21,26,30]. Any hospital wishing to calculate personal cost savings would need to make calculations based on their own ECV success and cesarean rates. Hospital stay, hence costs, are longer following cesarean and we did not calculate this benefit. Furthermore, cost of emergency cesarean delivery and the impact of spontaneous version were not calculated. Some women may refuse ECV, despite the possibility that ECV may be successful and allow them a vaginal delivery, or may have contraindications. Implementation of a formal decision management plan may increase ECV uptake [5]. Overheads (lighting, administration) are usually considered fixed costs and not itemized. Costs are calculated locally, and the overhead of 20% used in Israel may differ in other countries.

## Conclusions

Performing ECV using spinal anesthesia results in large cost savings to the hospital overall, since the rate of cesarean delivery can be reduced. The potential savings in other countries, where unit procedure (specifically manpower) costs are higher, may be larger than those in Israel. Protocols for advising patients of the benefits of ECV, and the additional benefits of anesthesia should be implemented. Both the patient and the hospital stand to gain financially and otherwise from successful ECV with subsequent vaginal delivery.

## Competing interests

The authors declare that they have no competing interests.

## Authors’ contributions

CFW and YE contributed to concept, design, analysis and preparation of the manuscript. PSS, YW and GG contributed to design, data analysis and content of the manuscript. All authors reviewed the final manuscript. All authors read and approved the final manuscript.
